# Gene- and Protein-Delivered Zinc Finger–Staphylococcal Nuclease Hybrid for Inhibition of DNA Replication of Human Papillomavirus

**DOI:** 10.1371/journal.pone.0056633

**Published:** 2013-02-20

**Authors:** Takashi Mino, Tomoaki Mori, Yasuhiro Aoyama, Takashi Sera

**Affiliations:** 1 Department of Synthetic Chemistry and Biological Chemistry, Graduate School of Engineering, Kyoto University, Kyoto, Japan; 2 Department of Applied Chemistry and Biotechnology, Graduate School of Natural Science and Technology, Okayama University, Okayama, Japan; 3 Department of Molecular Chemistry and Biochemistry, Faculty of Science and Engineering, Doshisha University, Kyoto, Japan; 4 Laboratory of Infection and Prevention, Institute for Virus Research, Kyoto University, Kyoto, Japan; The University of Queensland, Australia

## Abstract

Previously, we reported that artificial zinc-finger proteins (AZPs) inhibited virus DNA replication in planta and in mammalian cells by blocking binding of a viral replication protein to its replication origin. However, the replication mechanisms of viruses of interest need to be disentangled for the application. To develop more widely applicable methods for antiviral therapy, we explored the feasibility of inhibition of HPV-18 replication as a model system by cleaving its viral genome. To this end, we fused the staphylococcal nuclease cleaving DNA as a monomer to an AZP that binds to the viral genome. The resulting hybrid nuclease (designated AZP–SNase) cleaved its target DNA plasmid efficiently and sequence-specifically in vitro. Then, we confirmed that transfection with a plasmid expressing AZP–SNase inhibited HPV-18 DNA replication in transient replication assays using mammalian cells. Linker-mediated PCR analysis revealed that the AZP–SNase cleaved an HPV-18 *ori* plasmid around its binding site. Finally, we demonstrated that the protein-delivered AZP–SNase inhibited HPV-18 DNA replication as well and did not show any significant cytotoxicity. Thus, both gene- and protein-delivered hybrid nucleases efficiently inhibited HPV-18 DNA replication, leading to development of a more universal antiviral therapy for human DNA viruses.

## Introduction

Previously, we demonstrated that inhibition of the binding of a viral replication protein to its replication origin by using an artificial zinc-finger protein (AZP; [1]) is an effective tactic to prevent DNA virus infection in plants [2]. Then, we explored the feasibility of the methodology as a novel antiviral therapy for human DNA viruses. As a model system, we first applied AZP technology to human papillomavirus [3]. Papillomaviruses are double-stranded DNA viruses that induce benign proliferative squamous epithelial and fibroepithelial lesions (warts and papillomas) in their natural hosts. They have been isolated from a variety of animal species, and over 100 human papillomavirus (HPV) types have been identified and fully sequenced so far (reviewed in reference [4]). A subgroup of HPV classified as “high-risk” viruses, including HPV types 16, 18, 31, 35, 39, 45, 51, 52, 58, and 59, has been found to be associated with the development of cervical cancer [5], [6]. Each year, about 500,000 such infections of the uterine cervix undergo malignant conversion, making cervical cancer the second most common malignancy in women worldwide [7]. About 90% of such tumors contain high-risk HPVs, with HPV-16 and -18 being the most prevalent. The incidence shows no evidence of declining, and current treatment options are limited (http://boehringer-ingelheim.ca/research/res_area_humpap.asp). Therefore, effective antiviral therapies/treatments for this widespread and troublesome disease are clearly needed.

The papillomavirus proteins required for viral DNA replication are the viral E1 and E2 proteins (reviewed in reference [8]). The E1 protein is a 70–80 kDa nuclear phosphoprotein possessing DNA helicase activity (reviewed in reference [9]). Sequence-specific binding of E1 to the viral origin of replication is most likely mediated by the papillomavirus E2 protein [10]–[14]: E2 bound to the origin recruits E1 to the origin, which results in initiation of the replication process. Therefore, it is highly likely that one strategy for efficient inhibition of HPV replication is to block binding of E2 to its replication origin. To prove the concept, we designed AZPs blocking HPV type 18 (HPV-18) E2 binding [3]. In transient replication assays using the designed AZPs, we demonstrated that both gene- and protein-delivered AZPs efficiently inhibited HPV-18 DNA replication in mammalian cells [3], [15]. Although our AZP technology is effective for both plant and animal DNA viruses, the mechanism of DNA replication of viruses of interest must be unraveled in advance; at least, the DNA binding site of a viral replication protein should be sequenced.

Here, to strengthen AZP technology further, we explored the feasibility of inhibition of HPV-18 DNA replication by using AZP-based artificial endonucleases. One potential advantage of this approach is that the genome sequence information on DNA viruses of interest should be adequate for the application. Prior to our study, Horner and DiMaio reported the first application of an artificial endonuclease to cleave integrated HPV DNA in HeLa cervical carcinoma cells [16]. This group generated a chimeric nuclease (designated BEF in reference [16]) comprising the DNA binding domain of bovine papillomavirus type 1 (BPV1) E2 protein and the DNA-cleavage domain of a FokI endonuclease such as zinc-finger nucleases (ZFNs; [17]) that harbor an engineered zinc-finger protein as their DNA-binding domain and the FokI cleavage domain, and demonstrated clearly that the BEF nuclease effectively cleaved its target DNA in vitro and in HeLa cells. Although the adenovirus-delivered BEF nuclease induced cellular senescence in HeLa cells, molecular analysis revealed that the DNA-binding domain of the BPV1 E2 protein, but not the FokI domain of this nuclease, was responsible for senescence. One potential drawback to his approach is the use of a cloned viral DNA-binding protein, requiring information on the molecular structure of viruses of interest. This raises the questions of what a viral DNA-binding protein is, which region encodes its DNA-binding domain, and so on. Artificial DNA binding proteins such as AZPs [1], [18]–[20], zinc-finger arrays [21], [22], and transcription activator-like (TAL) effectors [23], [24] will increase the versatility of the nuclease technology for a DNA virus when the viral genome of interest has been sequenced and artificial DNA-binding proteins are precisely generated. During the course of our study, the application of ZFNs and TAL effector-FokI hybrids (TALENs), which are currently the most promising artificial endonuclease (reviewed in references [25]–[27]), to DNA viruses including HPV was proposed [22], [28].

In the present study, we generated a novel artificial endonuclease by fusing an AZP for HPV-18 to the staphylococcal nuclease (designated SNase), which cleaves DNA as a monomer. The resulting hybrid nuclease was designated AZP–SNase. Although the SNase is known to cleave single-stranded DNA and double-stranded DNA (dsDNA) as well as RNA [29], the SNase has been successfully used as a dsDNA-cleaving domain of artificial endonucleases in vitro [30], [31]. One advantage of AZP–SNase is that only one AZP is required to cleave target DNA, leading to more convenient design of artificial endonucleases or targeting. In contrast, because ZFNs and TALENs require to form the dimer through the FokI catalytic domain to cleave one target DNA [32], [33], we need to chose two adequate sites as their target DNA and construct two ZFN or TALEN molecules. In the present study, using AZP–SNase, we demonstrated that both transfection of a mammalian expression plasmid for the AZP–SNase and cell-permeable AZP–SNase efficiently inhibited HPV-18 DNA replication in transient replication assays. We also demonstrated that cell-permeable AZP–SNase did not exhibit any significant cytotoxicity in the transduced cell-lines even 12 days after transduction.

## Materials and Methods

### Plasmid constructions

The six-finger AZP used in the present study is the one (designated AZP_HPV_-1 in reference [3]) that did not efficiently reduce the replication of HPV-18 DNA in our previous transient replication assays, although the AZP sequence-specifically recognizes a 19-bp DNA, 5′-GAAAACGGTCGGGACCGAA-3′, with an apparent dissociation constant of 10 nM [3]. DNAs encoding the AZP and SNase were cloned stepwise into a modified pET-21a (Novagen) plasmid to prepare an *Escherichia coli* expression plasmid for the cell-permeable zinc-finger nuclease AZP–SNase–R9. The modified plasmid contains an N-terminal T7 tag, a C-terminal V5 tag, a nuclear localization signal (NLS) from the simian virus 40 large T antigen, a multi-cloning site for AZP and SNase, and a 9-mer of arginine (R9; [15], [34]) as a cell-penetrating peptide (CPP).

A mammalian expression plasmid for AZP–SNase, designated pCMV-AZP–SNase, was prepared by cloning the AZP and SNase open reading frames (ORFs) stepwise into a modified pcDNA3.1 (Invitrogen) plasmid. The modified plasmid contains the N-terminal T7 tag, C-terminal V5 tag, NLS, and a multi-cloning site for AZP and SNase. The six plasmids used for DNA cleavage and transient replication assays (described below), pRL-E1, pRL-E2, pOri-WT (designated pUC-Ori177 in reference [3]), pOri-MT1 (designated pUC-Ori177MT2 in reference [3]), pOri-MT2 (designated pUC-Ori177MT10 in reference [3]), and pCMV-AZP, were prepared as described previously [3].

### Protein overexpression and purification of AZP–SNase–R9

AZP–SNase–R9 was overexpressed in *E. coli* and purified essentially as previously described [1], except that harvested *E. coli* cells were lysed in 100 mM Tris-HCl, pH 8, 100 mM NaCl, 1 mM ZnCl_2_, and 5 mM DTT by sonication. The protein solution was clarified by centrifugation and loaded onto a Bio-Rex 70 column (Bio-Rad, Hercules, CA, USA), and each protein was then eluted with 600 mM NaCl buffer. The purified AZP–SNase–R9 protein was >95% homogeneous, as judged by sodium dodecyl sulfate-polyacrylamide gel electrophoresis. The protein concentration was determined using an ESL protein assay kit (Roche Molecular Biochemicals, Indianapolis, IN, USA).

### Cleavage of plasmid DNA by AZP–SNase–R9

A target DNA plasmid (5 nM) was incubated with 5 nM AZP–SNase–R9 at 4°C for 10 min in a reaction buffer (30 mM Tris-HCl, pH 7.5, 100 mM NaCl, and 1 µg µl^−1^ tRNA). A CaCl_2_ solution was then added to the mixture to a final concentration of 1 mM and incubated at 37°C for 5 min. After the cleavage reaction, the reaction mixture was extracted with phenol and digested with XmnI to distinguish between the DNA substrate and cleaved products more clearly. The final reaction mixtures were separated on 0.8% agarose gel. The gel was photographed under UV irradiation. These experiments were repeated independently three times.

### Identification of cleavage sites by AZP–SNase–R9

DNA fragments (200 bp) used to identify cleavage sites were prepared by PCR with primers labeled at the 5′ ends with Alexa680: primer set 1, labeled forward primer 5′-TTGTGGTGTGTTTCTCACATCTTTTATATA3′ and reverse primer 5′-AGCTATGACCATGATTACGCCAAGCTTGCA-3′; primer set 2, forward primer 5′-GTTGTAAAACGACGGCCAGTGAATTCGAGC-3′ and labeled reverse primer 5′-CCGAAATAGGTTGGGCAGCACATACTATAC-3′; pOri-WT as a template. The dsDNA (0.1 µg) containing the 5′-end-labeled sense or antisense strand was treated with AZP–SNase–R9 (50 nM) as in cleavage of plasmid DNA. After phenol extraction, the DNA pellet was resuspended in formamide and separated on 6% denaturing polyacrylamide gel together with DNA makers treated with Maxam-Gilbert's C+T reaction [35]. The gel was analyzed by using Odyssey (Aloka, Tokyo, Japan). The experiment was repeated independently three times.

### Transient replication assays

Transient replication assays were performed essentially as previously described [3]. A total of 8×10^5^ cells of the human cell line 293H (Invitrogen) were plated onto a BioCoat poly-D-lysine 12-well plate (Becton Dickinson) and maintained in Dulbecco's modified Eagle's medium (Invitrogen) supplemented with 0.1 mM nonessential amino acids and 10% fetal bovine serum (Invitrogen). Three plasmids necessary for transient replication, pRL-E1 (1.5 µg), pRL-E2 (0.17 µg), and pOri derivative (0.17 µg), were cotransfected with pCMV-AZP–SNase, pCMV-AZP, or a vacant plasmid, pcDNA3.1 (0.17 µg) by using Lipofectamine 2000 (Invitrogen) according to the protocol accompanying the reagent. Three days after transfection, low-molecular-weight DNA was isolated by Hirt extraction [36]. The samples were first treated with HindIII to linearize them. To distinguish between replicated and unreplicated DNAs, one-half of each sample was then treated with an excess of DpnI to remove the unreplicated, methylated input DNA [37]. DpnI resistance has been used to demonstrate DNA replication in studies with mammalian cells, including studies with HPVs [38], [39]. One percent of the remaining half of each linearized sample was used to confirm that equal amounts of the plasmids used for each transient replication assay were introduced into 293H cells. The DNA samples were separated by electrophoresis (in 0.8% agarose gels with 0.5×Tris-borate-EDTA buffer), followed by Southern blot hybridization.

A 200-bp digoxygenin (DIG)-labeled probe specific to an ampicillin-resistance gene was prepared from pUC-19 by PCR using DIG-11-dUTP (Roche Molecular Biology) and the primer set 5′-CGGCATCAGAGCAGATTGTACTGAGAGTGC-3′ and 5′-TACCCAACTTAATCGCCTTGCAGCACATCC-3′. Because pRL-E1, pRL-E2, pCMV-AZP, and pOri derivatives contain the ampicillin-resistance gene as a selection marker, all these plasmids could be detected by using the DIG-labeled probe. DNAs were resolved in a 0.8% agarose gel and transferred onto a Nytran SuPerCharge membrane by use of a TurboBlotter (Schleicher & Schuell, Dassel, Germany). After hybridizing with the DIG-labeled probe, DNA bands corresponding to the plasmids used for transient replication assays were recorded on X-ray films, using anti-DIG-AP and CDP-Star according to the accompanying protocols (Roche Molecular Biology). DNA band intensities on X-ray films were digitized and quantitated using UN-SCAN-IT (Silk Scientific, Inc., Orem, UT, USA). Average DNA band intensities of replicated pOri derivatives were calculated from at least three independent experiments and normalized with DNA band intensities of input pRL-E1 or pOri derivative.

### Ligation-mediated PCR (LM-PCR) of DNA double-strand breaks in 293H cells by AZP–SNase

Each Hirt-extracted DNA sample from the transient replication assays described above was treated with T4 polynucleotide kinase to remove 3′-phosphoryl groups generated by AZP–SNase cleavage [29] and added 5′-phosphates at 37°C for 30 min, and then treated with T4 DNA polymerase to blunt staggered DNA lesions at 12°C for 15 min. After phenol extraction, the blunted DNA was ligated with DNA Ligation Kit Ver.2.1 (Takara, Shiga, Japan) to 100 pmol of a double-stranded blunt-ended linker, which was generated by annealing 5′-GCGGTGACCCGGGAGATCTGAATTC-3′ and 5′-GAATTCAGATC-3′ [40], in a total volume of 10 µl overnight at 16°C. The ligated DNA sample (0.5 µl) was amplified by PCR with the primer set of the linker-specific primer 5′-GCGGTGACCCGGGAGATCTGAATTC-3′ and biotin-labeled pOri-specific primer 5′-CAGCTGGCACGACAGGTTTCCCGACTGGAA-3′. The PCR conditions were as follows: initial denaturation at 94°C for 3 min and 10 cycles of heating (94°C, 30 s), annealing (66°C, 2 min), and elongation (72°C, 1 min). The PCR product was purified by using a QIAGEN PCR purification kit (Qiagen, Valencia, CA, USA) and further purified with Dynabeads M-270 Streptavidin (Invitrogen, Carlsbad, CA, USA) according to the protocol accompanying the reagent. The purified sample was used as the template for a second round of PCR with the primer set of the linker-specific primer and pOri-specific primer. The PCR conditions were initial denaturation at 94°C for 3 min and 30 cycles of heating (94°C, 30 s), annealing (66°C, 2 min), and elongation (72°C, 1 min). The reaction mixtures were separated on 2% agarose gel. The gels were photographed under UV irradiation. The experiment was repeated independently three times.

### Analysis of AZP–SNase–R9 cytotoxicity

The cytotoxicity of AZP–SNase–R9 was evaluated by an MTT (3-(4,5-dimethylthiazol-2-yl)-2,5-diphenyltetrazolium bromide) assay. A total of 6×10^4^ of 293H cells were plated onto a BioCoat™ poly-D-lysine 96-well plate (Becton Dickinson, Franklin Lakes, NJ, USA) and maintained in 0.1 ml of Dulbecco's modified Eagle's medium supplemented with 0.1 mM nonessential amino acids and 10% fetal bovine serum. An AZP–SNase–R9 solution (10 µl in Opti-MEM) was added to the culture medium. After incubation for 3 days, 10 µl of the solution from a Cell Counting Kit-8 (Dojin, Tokyo, Japan) was added to each well, and the plate was incubated at 37°C for 2 h. The cytotoxicity of AZP–SNase–R9 was evaluated by measuring the absorbance at 450 nm with an ARVO SX 1420 multilabel counter (Wallac, Freiburg, Germany). The experiments were carried out in triplicate and repeated independently three times.

The cytotoxicity of AZP–SNase–R9 12 days after transduction was also evaluated. In this experiment, the final concentration of AZP–SNase–R9 added to the culture medium was 80 pM. After addition of AZP–SNase–R9, the transduced 293H cells were incubated for 3 days. Then, cells were collected by trypsinization. One-third or one half of the collected cells were plated and incubated for 3 days. The passage procedure was repeated two more times. Finally, the cytotoxicity of AZP–SNase–R9 in the daughter cells of the fourth generation was evaluated as described above. The experiments were carried out in triplicate and repeated independently five times.

## Results

### Design and generation of cell-permeable zinc-finger nuclease AZP–SNase–R9 for protein delivery

**Figure 1:**
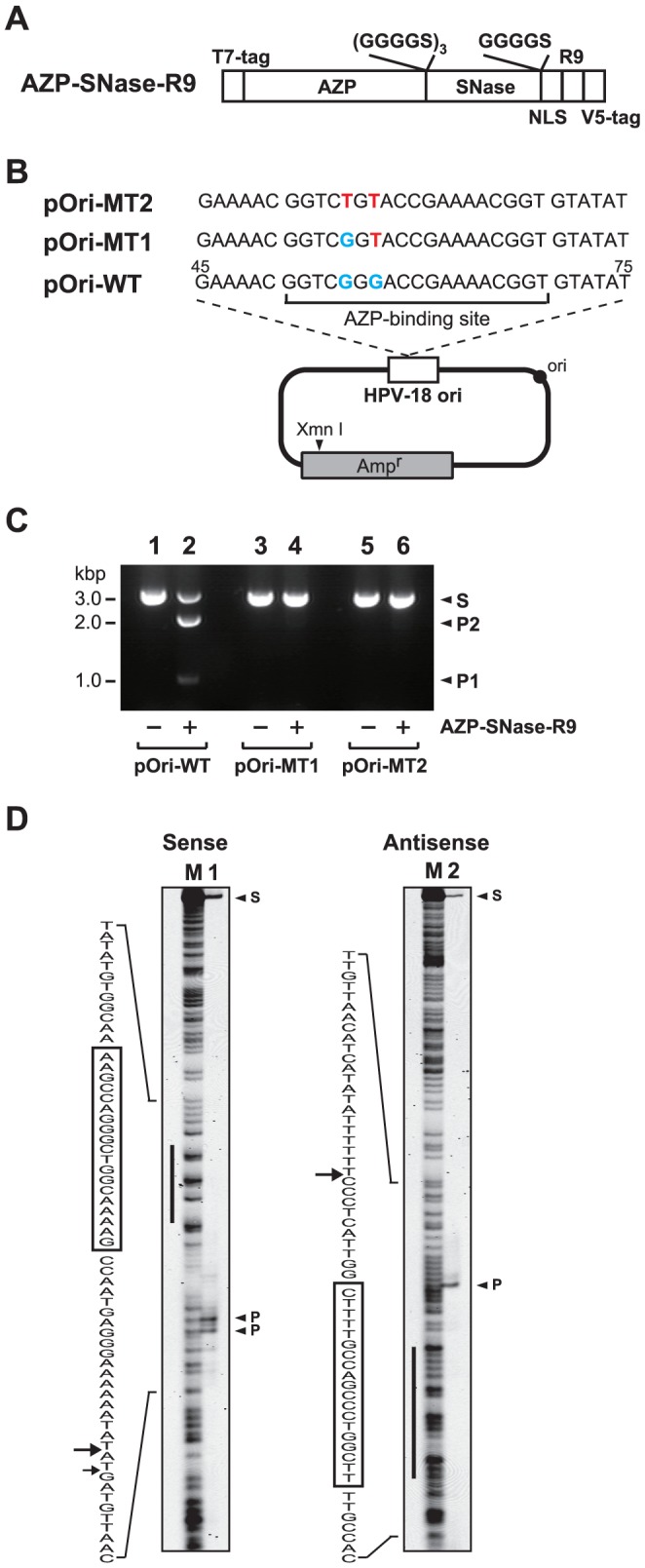
In vitro DNA cleavage by AZP–SNase–R9. (A) Schematic representation of a cell-permeable AZP-SNase, AZP–SNase–R9. R9 as a CPP was fused to a six-finger AZP containing an SNase cleaving domain, NLS, and T7 and V5 tags as epitope tags to yield AZP–SNase–R9. The amino acids shown above the open boxes are linker peptides. (B) Wild-type and mutant HPV-18 *ori* plasmids used in this study. The open box indicates the 177-bp HPV-18 replication origin (HPV-18 nt 7800 to 7857 and 1 to 119). The red letters indicate mutations of the AZP-binding site in the HPV-18 replication origin. The numbers above the DNA sequence indicate their locations (in nt) in the HPV-18 genome. (C) AZP–SNase–R9 distinguished a difference of one-bp. Treatment of wild-type or mutant target plasmids (5 nM) by AZP–SNase–R9 (5 nM) at 37°C for 5 min, followed by XmnI digestion. The final reaction mixtures were analyzed on 0.8% agarose gel. Lanes 1, 3, and 5, none; lanes 2, 4, and 6, AZP–SNase–R9. Target plasmids used are indicated below each lane. S, a 2.9-kbp DNA substrate; P1, a 0.9-kbp cleavage product; P2, a 2.0-kbp cleavage product. (D) Cleavage of 5′-end-labeled 200-bp dsDNA targets by AZP–SNase–R9. After reaction with AZP–SNase–R9 at 37°C for 5 min, the reaction mixtures were separated on 6% denaturing gel. Black bars next to the C+T marker lane (M) indicate the binding site of the AZP used. Boxes indicate DNA sequence of the binding site of the AZP, and the main cleavage sites were indicated by black arrows. S: DNA substrate, P: DNA cleavage products.

To examine the feasibility of inhibition of HPV-18 replication by our novel zinc-finger nuclease, we used a previously reported six-finger AZP [3], as the DNA-binding domain. The AZP was designed using our nondegenerate recognition code table [1] to prevent E2, a replication protein of HPV-18, from binding to its replication origin, leading to inhibition of HPV-18 DNA replication [3]. Although the AZP had a great affinity toward its binding site (its apparent dissociation constant: 10 pM), the tight binding alone was not enough to inhibit HPV-18 DNA replication [3]. In the present study, to prove our concept, the AZP was fused with SNase as a DNA-cleavage domain and R9 as a CPP to generate cell-permeable AZP–SNase. The CPP was chosen because it exhibited greater cell penetration than TAT_47–57_ in our and others' studies [34], [41], [42]. The R9 derivative AZP–SNase–R9 contains a T7 tag, the AZP, SNase, an NLS from the SV40 large T antigen, R9, and a V5 tag from the N-terminus to the C-terminus (see Figure 1A). AZP–SNase–R9 was expressed in *E. coli* for purification (data not shown), and the purified AZP–SNase–R9 was used for the following experiments.

### In vitro DNA cleavage by AZP–SNase–R9

First, we examined DNA cleavage of HPV-18 *ori* plasmids (Figure 1B) by AZP–SNase–R9 in vitro. As shown in lane 2 of Figure 1C, AZP–SNase–R9 efficiently cleaved its wild-type target plasmid pOri-WT with no side product. To confirm the site-specific cleavage by AZP–SNase–R9, we cleaved mutant target plasmids. The mutant plasmids pOri-MT1 and pOri-MT2 contain one and two mutations, respectively, in the AZP-binding site (Figure 1B). In contrast to cleavage of the WT target plasmid (Figure 1C, lane 2), no site-specific cleavage product by AZP–SNase–R9 was observed in mutant target plasmids (Figure 1B, lanes 4 and 6).

Next, we identified the cleavage sites of AZP–SNase–R9 (Figure 1D). Five-prime-end-labeled 200-bp DNA probes containing the 19-bp target recognized by AZP–SNase–R9 were digested by AZP–SNase–R9, and then these products were separated on 6% denaturing gel. Comparison with a DNA (C+T) marker generated by the Maxam-Gilbert method demonstrated that AZP–SNase–R9 cleaved the target DNA mainly at one site of both strands, producing 9-base sticky ends (Figure 1D).

### Design and generation of AZP–SNase for gene delivery

**Figure 2:**
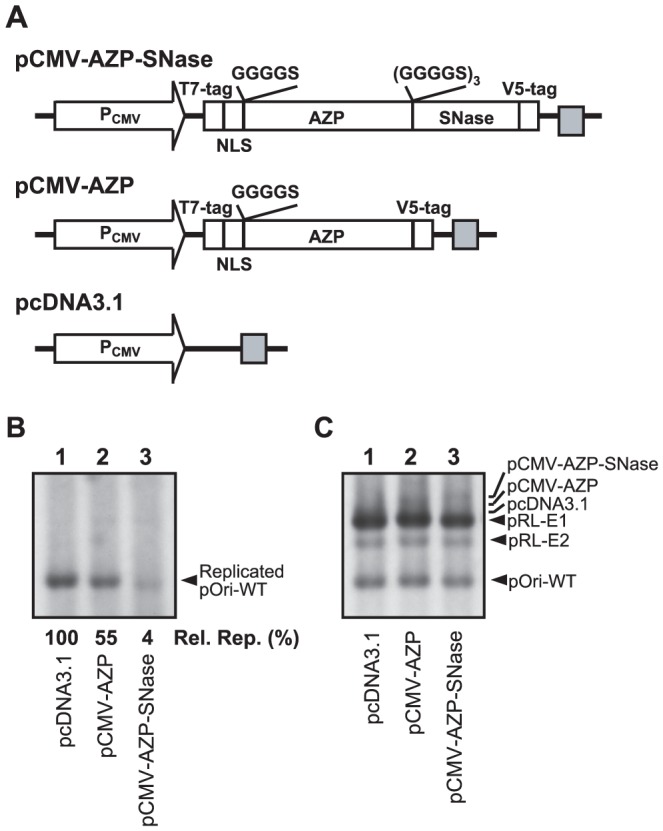
Transient replication of HPV-18 DNA with gene-delivered AZP–SNase. (A) Plasmids for expression of AZP derivatives used in this study. The open arrows and grey boxes indicate a CMV promoter and a BGH polyadenylation signal, respectively. The open boxes indicate open reading frames of a T7 tag, NLS, the same AZP described in Figure 1A, SNase, and a V5 tag, but do not reflect actual sizes. The amino acids shown above the open boxes are linker peptides. (B) Inhibition of HPV-18 DNA replication by gene-delivered AZP–SNase. Transient replication assays were performed with pRL-E1 for E1 expression, pRL-E2 for E2 expression, and pOri-WT as the HPV-18 *ori* plasmid along with expression plasmids for the AZP expression plasmid or the vacant plasmid pcDNA3.1 described below each lane. Hirt-extracted samples were digested with DpnI to eliminate unreplicated input plasmids (see Materials and Methods). Inhibition efficiencies of DNA replication by AZP derivatives were judged by comparison of relative amounts of pOri-WT. The amount of replicated pOri-WT in the presence of each AZP derivative was compared with that in the absence of AZP (100%). The average data [Rel. Rep. (%)] from at least three independent assays are indicated below each lane. (C) Examination of amounts of plasmids introduced into 293H cells for panel B. This panel shows Southern blot hybridization with Hirt-extracted DNA samples which were not treated with DpnI, indicating relative amounts of plasmids introduced into 293H cells.

Because the AZP–SNase–R9 sequence-specifically recognized and efficiently cleaved its target plasmid near the binding site in vitro, we next investigated whether the zinc-finger nuclease repressed or inhibited HPV-18 DNA replication in mammalian 293H cells by cleaving the HPV-18 *ori* plasmid. To this end, we constructed a mammalian expression plasmid encoding the zinc-finger nuclease. In the designated plasmid pCMV-AZP–SNase, the AZP–SNase ORF was placed under the control of the human cytomegalovirus promoter (Figure 2A). Additionally, two plasmids, pCMV-AZP–SNase and pcDNA3.1, were used as a control (Figure 2A).

### Inhibition of HPV-18 DNA replication by gene-delivered AZP–SNase

HPV DNA replication can be transiently reconstituted in mammalian cells by using mammalian expression plasmids for viral E1 and E2 proteins and a plasmid containing an HPV replication origin (HPV *ori* plasmid). The DNA replication mechanism has been extensively investigated by using transient replication assays (for examples, see references [11], [43], [44]. These experiments revealed that the E2 protein plays a critical role in replication by recruiting the E1 helicase to the replication origin, suggesting that blocking E2 binding is an attractive approach to inhibition of HPV DNA replication. Indeed, the gene-delivered AZP (used in the present study) designed to block HPV-18 E2 binding reduced the replication to 12% in transient replication assays [3]. In the transient replication assays that we used, we experimentally confirmed that expression of E2 is indispensable for the HPV-18 DNA replication [3]. In the present study, we used the same assays to evaluate gene-delivered AZP–SNase first. In these assays, an input HPV-18 *ori* plasmid (designated pOri-WT) prepared in *E. coli* containing a dam methylase was eliminated after transient replication by DpnI, which digests hemi- and homomethylated 5′-GATC-3′ sequences, and the amounts of the replicated pOri-WT alone were quantitated by Southern blot analysis [11], [44]. As shown in Figure 2B, gene delivery of AZP–SNase reduced the replication level to 4%±2% of that of a control experiment with a vacant plasmid pcDNA3.1. In contrast, the AZP alone did not efficiently repress HPV-18 DNA replication as previously reported [3]. In the transient replication assays, the same amounts of plasmids used for the assays were introduced into 293H cells, as judged from Southern blot analysis with Hirt-extracted DNA samples untreated with DpnI (Figure 2C).

### Specific binding of AZP–SNase to HPV-18 DNA replication origin inhibits DNA replication

The results described above suggest that specific binding of AZP–SNase to the HPV-18 replication origin inhibits the DNA replication. To examine this further, we conducted transient replication assays by using plasmids with mutant replication origins. If no repression of DNA replication of mutant *ori* plasmids, whose mutations impair AZP binding and, as a result, DNA cleavage by AZP–SNase (see Figure 1C), was observed in the presence of AZP–SNase, this would demonstrate that the inhibition of DNA replication of the wild-type HPV-18 *ori* shown in Figure 2B was caused by specific binding of the designed AZP–SNase to the replication origin; however, if effective inhibition of mutant *ori* replication was observed even in the presence of AZP–SNase, then the cause of the inhibition of DNA replication of the wild-type HPV-18 *ori* shown in Figure 2B was an unknown non-specific binding of AZP–SNase. For this experiment, two mutant *ori* plasmids were used (see Figure 1B). These mutant *ori* plasmids, pOri-MT1 and pOri-MT2, contained one and two mutations, respectively, in the binding site of AZP–SNase (Figure 1B). We note that in these mutant *ori* plasmids used, mutations were introduced into a spacer region (i.e., 5′-CGGG-3′) between two E2-binding sites (5′-ACCGAAAACGGT-3′) to minimize the effect of these mutations on E2 binding, whereas replication levels of these mutant *ori* plasmids were lower than that of the wild type *ori*, pOri-WT [3]. It has been reported that mutation of the spacer region also reduces the replication level significantly [45].

**Figure 3:**
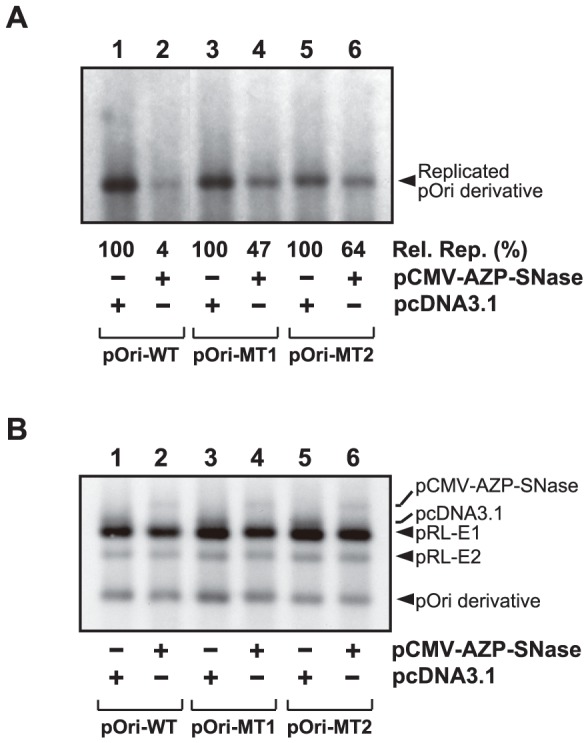
Transient replication of HPV-18 mutant *ori* plasmids with gene-delivered AZP–SNase. (A) Replication of HPV-18 mutant *ori* plasmids in the presence of pCMV- AZP–SNase. Transient replication was assayed with pRL-E1, pRL-E2, and a pOri-WT derivative along with pCMV-AZP–SNase or pcDNA 3.1, as indicated below each lane. Hirt-extracted samples were digested with DpnI to eliminate unreplicated input plasmids (see Materials and Methods). Efficiencies of inhibition of DNA replication by AZPs were judged by comparison with relative amounts of each pOri derivative. The amount of each replicated pOri derivative in the presence of AZP–SNase was compared with that in the absence of AZP (100%). The average data [Rel. Rep. (%)] from three independent assays are indicated below each lane. (B) Examination of amounts of plasmids introduced into 293H cells for panel A. This panel shows Southern blot hybridization with Hirt-extracted DNA samples which were not treated with DpnI, indicating relative amounts of plasmids introduced into 293H cells.

Transient replication assays with these two mutant *ori* plasmids (together with the wild type *ori* plasmid) were carried out according to the procedure described for Figure 2. As shown in Figure 3A, lanes 3 and 4, one mutation reduced the inhibition by AZP–SNase to 47%±3%, indicating that AZP–SNase recognized a one-bp difference in 293H cells too. Moreover, the two mutations attenuated inhibition of DNA replication by AZP–SNase (Figure 3A, lane 6) to 64%±6% of that of the control (Figure 3A, lane 5). These results demonstrate that AZP–SNase discriminates differences of a couple of base pairs in 293H cells. In the transient replication assay with AZP–SNase for each mutant *ori* plasmid, the same amounts of plasmids as those used in the assay with pcDNA3.1 were introduced into 293H cells, as shown in Figure 3B (for example, compare lane 3 with lane 4). Because half-maximal binding to the mutant probes was observed at concentrations of AZP that were >10-fold higher than that for the wild-type probe [3], the loss of inhibition of replication of these mutant *ori* plasmids indicates that specific binding of AZP–SNase to the HPV-18 replication origin inhibited DNA replication.

### Site-specific cleavage of HPV-18 DNA in 293H cells by gene-delivered AZP–SNase

**Figure 4:**
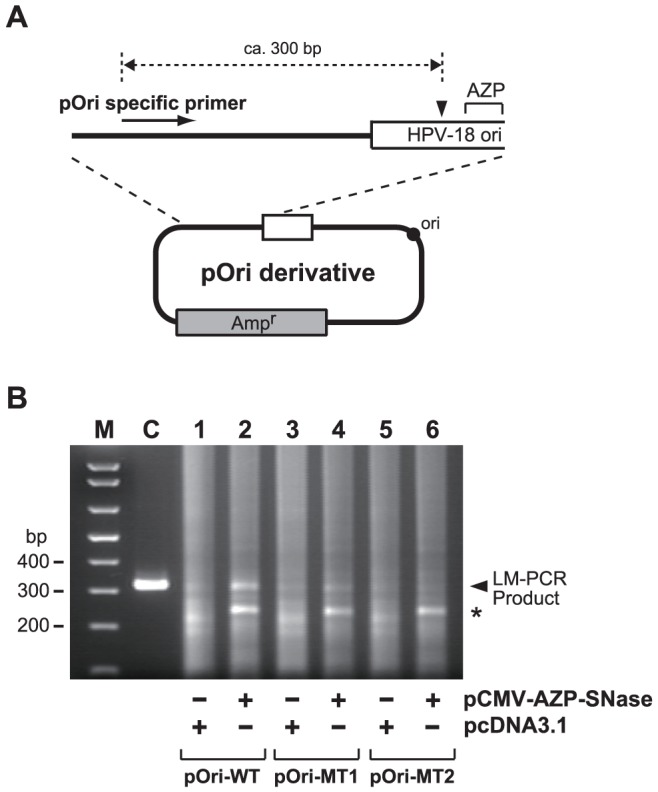
Cleavage of the pOri-WT plasmid around AZP-binding site in 293H cells by AZP–SNase. (A) Schematic representation of the location of the pOri specific primer used for LM-PCR on pOri derivatives The black triangle indicates the main site of cleavage by AZP–SNase. The expected LM-PCR product of cleavage by AZP–SNase is around 300 bp. The drawing is not to scale. (B) LM-PCR analysis of Hirt-extracted DNA samples after transient replication assays. Hirt-extracted DNA samples after the transient replication assays in the presence of pCMV-AZP–SNase (Figure 3A) were blunt-ended, ligated to a blunt-ended linker, and then LM-PCR was performed with the pOri specific primer and linker primer (see Materials and Methods). Lane M: a DNA size marker (TrackIt 1 kb Plus DNA Ladder), lane C: a marker of an expected LM-PCR product derived from cleavage by AZP–SNase. The identity of the additional band (indicated with an asterisk) is described in the Discussion).

In the transient replication assays described above, we demonstrated that gene-delivered AZP–SNase efficiently inhibited HPV-18 DNA replication by binding to its target site in 293H cells. However, no direct evidence that DNA cleavage by AZP–SNase caused the inhibition in 293H cells was in hand. Therefore, we conducted LM-PCR using Hirt-extracted DNA samples obtained from the transient replication assays described above. LM-PCR analysis enables estimation of locations of genomic DNA lesion [40]. In the analysis, genomic DNA ends generated by a DNA lesion were converted to blunt ends, ligated to a blunt-ended linker adaptor, and then amplified by PCR with a genome-specific primer. Due to the length of the LM-PCR products, the location of a DNA lesion relative to the genome-specific primer used can be estimated. In the present study, a pOri specific primer (Figure 4A) was used instead of a genome specific primer to evaluate cleavage of pOri-WT by AZP–SNase in 293H cells. When a Hirt-extracted DNA sample obtained from a transient replication assay with the wild-type *ori* plasmid pOri-WT (Figure 3A, lane 2) was used as a template for LM-PCR, a PCR product (ca. 300 bp) corresponding to cleavage of the wild-type *ori* plasmid around its binding site by AZP–SNase was clearly observed (Figure 4B, lane 2). However, in the LM-PCR using a Hirt-extracted DNA sample obtained from a transient replication assay with the mutant *ori* plasmid pOri-MT1 harboring a one-bp mutation (Figure 3A, lane 4), the 300-bp DNA band intensity was reduced, as shown in lane 4 of Figure 4B. Additionally, in the LM-PCR using Hirt-extracted DNA sample obtained from transient replication assay with the mutant *ori* plasmid pOri-MT2 harboring a two-bp mutation (Figure 3A, lane 6), no detectable 300-bp DNA band was observed (Figure 4B, lane 6). Note that the 300-bp LM-PCR product was not detected in all any control experiment in the absence of AZP–SNase using the wild-type and mutant *ori* plasmids (Figure 4B, lanes 1, 3, and 5). This result demonstrated again that AZP–SNase discriminates differences of a couple of bp in 293H cells.

### Inhibition of HPV-18 DNA replication by protein-delivered AZP–SNase

**Figure 5:**
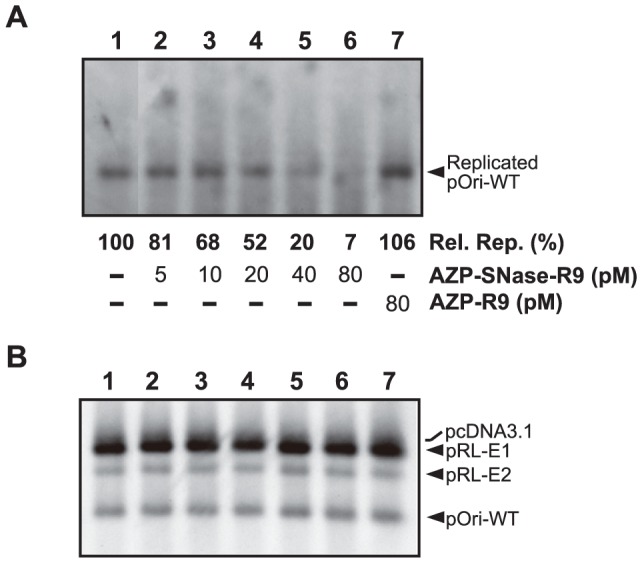
Transient replication of HPV-18 DNA with cell-permeable AZP–SNase–R9. (A) Inhibition of HPV-18 DNA replication by AZP–SNase–R9 or AZP–R9 as a control. The transient replication assays were performed with pRL-E1, pRL-E2, and pOri-WT along with AZP–SNase–R9 or AZP–R9 as indicated below each lane. Hirt-extracted samples were digested with Dpn I. Inhibition efficiencies of DNA replication by AZP–SNase–R9 or AZP–R9 were judged by comparison of relative amounts of pOri-WT. The amount of pOri-WT replicated in the presence of AZP–SNase–R9 or AZP–R9 was compared with that in the absence of the AZP derivatives (100%). The relative replication [Rel. Rep. (%)] from at least three independent assays is indicated below each lane. (B) Examination of amounts of plasmids introduced into 293H cells for panel A. This panel shows Southern blot hybridization with Hirt-extracted DNA samples which were not treated with DpnI, indicating relative amounts of plasmids introduced into 293H cells.

After we demonstrated that the gene-delivered AZP–SNase inhibited HPV-18 DNA replication in 293H cells by cleaving HPV-18 *ori* plasmids, we next investigated whether protein-delivered AZP–SNase inhibited HPV-18 DNA replication. For this experiment, transient replication assays with the wild type *ori* plasmid were carried out according to the procedure described for Figure 2. As shown in Figure 5A, cell-permeable AZP–SNase–R9 reduced the HPV-18 DNA replication level in a concentration-dependent manner: 80 pM AZP–SNase–R9 reduced the replication level to 7%±1% of that of a control experiment with no AZP–SNase–R9. In the transient replication assays, the same amounts of plasmids used for the assays were introduced into 293H cells, as judged from Southern blot analysis with Hirt-extracted DNA samples untreated with DpnI (Figure 5B).

### Cytotoxicity of AZP–SNase–R9

**Figure 6:**
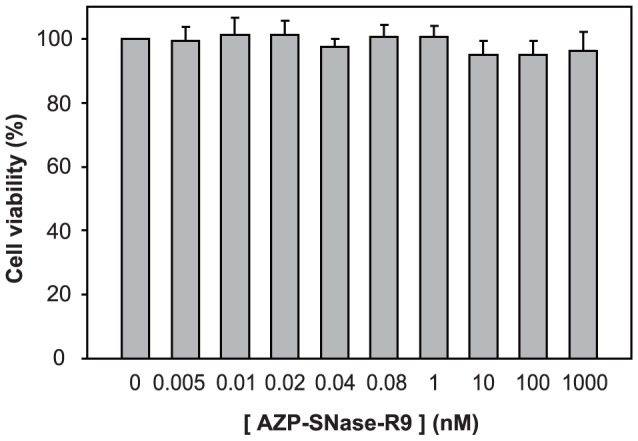
Cytotoxicity of AZP–SNase–R9. Three days after addition of AZP–SNase–R9 (0 to 1000 nM) to the culture medium, amounts of a water-soluble formazan dye generated by cellular enzymes were measured based on absorbance at 450 nm (OD_450_), and then cell viabilities (%) were calculated from the ratio of OD_450_ in the presence of AZP–SNase–R9 to OD_450_ in the absence of AZP–SNase–R9 as the control (100%). Concentrations of AZP–SNase–R9 used are indicated below each lane. The data represent an average of three independent experiments, and the SD is shown.

Finally, we investigated the cytotoxicity of AZP–SNase–R9 in a modified MTT assay with a highly water-soluble disulfonated tetrazolium salt [46], which is commercially available as a Cell Counting Kit-8 from Dojindo. In this assay, the cytotoxicity of a drug of interest is based on absorbance at 450 nm of the corresponding water-soluble formazan dye produced by cellular dehydrogenases. As shown in Figure 6, the cell viability in the presence of 80 pM AZP–SNase–R9, which reduced HPV-18 DNA replication to 7%, was 100.6%±3.9%. The cell viability in the presence of 1 nM AZP–SNase–R9 was 100.5%±3.7%. Moreover, even 1 µM AZP–SNase–R9, which is the highest concentration that we examined in this study, did not show any significant reduction of cell viability three days after transduction.

Furthermore, we investigated the cytotoxicity of AZP–SNase–R9 12 days after transduction with 80 pM AZP–SNase–R9, which reduced the replication level to 7%±1% of that of a control experiment with no AZP–SNase–R9 (Figure 5A) because incubation for three days after transduction might be too short for cytotoxicity of AZP–SNase to emerge. After addition of AZP–SNase–R9 to a final concentration of 80 pM, the transduced cells were incubated for three days. Then, the resulting cells were passaged three times. The cell viability of the daughter cells of the fourth generation was 97.4%±4.0% and not significantly different from that of a control experiment with no AZP–SNase–R9 (P = 0.22 by Student's *t*-test), indicating that 80 pM AZP–SNase–R9 did not show any significant cytotoxicity even 12 days after transduction. And we did not observe any detectable phenotypic change (e.g., the shape and growth rate of cells) of the daughter cells (data not shown).

## Discussion

In the present study, we demonstrated that our unique artificial endonuclease AZP–SNase cleaved an HPV-18 *ori* plasmid and discriminated a one-bp difference in vitro (Figure 1C). The gene-delivered AZP–SNase also inhibited HPV-18 DNA replication in transient replication assays (Figure 2B). When a gene was delivered to mammalian cells, inhibition of HPV-18 DNA replication by AZP–SNase was attenuated with an increasing number of mutations of the AZP-binding site in the HPV-18 *ori* plasmid (Figure 3A). These results clearly indicate that specific binding of AZP–SNase to the HPV-18 replication origin inhibited DNA replication in living 293H cells. Cleavage of the HPV-18 *ori* plasmid by AZP–SNase should have inhibited HPV-18 DNA replication because the endonuclease cleaved site-specifically in vitro (Figure 1D). However, in the above transient replication assays, no significant decrease of input *ori* plasmid was observed even in the presence of AZP–SNase (for example, compare lane 3 with lane 1 in Figure 2C). It is highly likely that the majority of input DNA plasmids introduced into 293H cells remained in the cytoplasm because DNA trafficking from the cytoplasm to the nucleus is an inefficient process [47], [48]. For example, as described by Wolff's group [47], direct cytoplasmic injection of approximately 10^7^ copies of a β-galactosidase reporter gene into 3T3 fibroblasts results at most in 1% of cells expressing the foreign gene. The HPV-18 *ori* plasmid trafficked into the nucleus seemed to be cleaved by AZP–SNase, leading to reduction of the replication of HPV-18 DNA to 4% of that of a control (lane 3 of Figure 2B). Indeed, as shown in lane 2 of Figure 4B, LM-PCR analysis of the Hirt-extracted DNA sample obtained from a transient replication assay with the wild-type *ori* plasmid revealed the sequence-specific DNA cleavage by AZP–SNase harboring an NLS. The LM-PCR product was not observed in the Hirt-extracted DNA sample obtained from a transient replication assay with the mutant *ori* plasmid having a two-bp mutation (Figure 4B, lane 6).

We should note that an additional DNA band (indicated by an asterisk in Figure 4B) was observed in the LM-PCR analysis. The approximately 250-bp PCR product was observed consistently in LM-PCR with mutant *ori* plasmids as well. The origin of the product remains to be unknown at this stage. It may be an artifact or be caused by an additional or non-specific cleavage by AZP–SNase. In in vitro DNA cleavage experiments, no additional DNA cleavage corresponding to the shorter LM-PCR product was observed. For example, no additional DNA cleavage was observed in Figure 1C. If AZP–SNase recognized and cleaved an additional site different from the original AZP-binding site, the cleavage product should have been observed in the experiments with the HPV-18 *ori* mutant plasmids. However, no detectable DNA cleavage product was observed in the experiments with pOri-MT1 and pOri-MT2 as shown in lanes 4 and 6 of Figure 1C.

We previously reported a zinc-finger-based artificial transcription factor fused to a CPP as the first example of a designed regulatory protein (DRP; [42]). After that report, we developed another DRP as the protein-based inhibitor of HPV-18 DNA replication [15], in which an AZP designed to block the HPV-18 E2 protein from binding to its replication origin was fused to an R9 CPP (designated AZP–R9). In transient replication assays, AZP–R9 showed a higher selectivity index or safety index (SI) (>300), defined as the ratio of the 50% inhibition concentration (IC_50_)/50% effective concentration (EC_50_), than the chemical cidofovir (SI = 15–42 in reference [49]) [15]. In the present study, we presented AZP–SNase–R9 as the third example of a DRP. The SI of AZP–SNase–R9 was at least 5×10^6^ because the EC_50_ was <20 pM (from Figure 5A) and the IC_50_ was ≫1 µM (from Figure 6). Accordingly, addition of the SNase cleavage domain to AZP–R9 enhanced the SI value >10,000-fold compared to AZP–R9 alone. Although we worried about the cytotoxicity of AZP–SNase–R9 due to the catalytic SNase domain, even 1 µM AZP–SNase–R9, which was the highest concentration that we examined in this study, did not show any significant cytotoxicity three days after transduction (Figure 6).

In the present study, we developed a novel artificial endonuclease by fusing SNase to a single AZP. However, we worried that AZP–SNase might bring unfavorable side effects to 293H cells due to potential off-target DNA cleavage and RNA cleavage. Because ZFNs and TALENs cleave target DNA as a dimer, the off-target DNA cleavage by a ZFN/TALEN monomer could be reduced. However, even in the case of ZFNs/TALENs, off-target DNA cleavage remains to be a critical issue [50], [51]. Therefore, we investigated the cytotoxicity of AZP–SNase–R9 not only in the first generation of the transduced 293H cells (Figure 6), but also in the fourth generation. We hypothesized that viable cells should have been significantly decreased in the fourth generation of cells transduced with AZP–SNase if AZP–SNase randomly cleaved genomic DNA in 293H cells due to off-target cleavage. As described in the Results section, the cellular viability (97.4%±4.0%, P = 0.22 by Student's *t*-test) was not significantly different from that of a control experiment with no AZP–SNase–R9 in the fourth generation of the transduced cell-lines. We did not observe any detectable phenotypic change (e.g., the shape and growth rate of cells) in the fourth generation of the transduced cell-lines (data not shown). Therefore, these results indicate that AZP–SNase did not impair genes (or genomic sites) critical for the maintenance of cellular homeostasis although we could not completely rule out the possibility of off-target DNA cleavage at this stage. Furthermore, although AZP–SNase should possess an RNA-cleaving activity due to the SNase domain, AZP–SNase neither showed any significant cytotoxicity nor caused any phenotypic change even in the fourth generation of the transduced cells, which indicats that AZP–SNase did not impair RNA critical for the maintenance of cellular homeostasis. Because AZP–SNase was fused to an NLS in the present study, AZP–SNase might not efficiently cleave mature RNA in the cytoplasm. Conversely, when the SNase is fused to an RNA-binding protein alone (i.e., with no NLS), the resulting fusion protein may serve as an unique molecular tool to specifically cleave target RNA and be used for RNA viruses as a novel antiviral therapy. In any case, at the next step, we will need to investigate the potential adverse effects in detail using animal models prior to the application of this technology to future clinical trial.

Finally, we note that during the course of our study, Barbas's group reported that ZFNs alone are amazingly cell-permeable and can be used to modify genetic information in living mammalian cells [52]). Although ZFNs alone were not directly compared with CPP-conjugated ZFNs in his study, the comparison will become more important for the development of cell-permeable ZFNs as a new molecular tool.

In summary, we demonstrated in transient replication assays that our unique artificial endonuclease AZP–SNase hybrid, which was delivered both in an expression plasmid and as a cell-permeable protein to mammalian cells, inhibited DNA replication of HPV-18, an important therapeutic target for cervical cancer, by cleaving the HPV-18 *ori* plasmid. We hope that this work will lead to the development of more versatile antiviral therapies and protein drugs for human DNA viruses.
